# Synthesis of silymarin–selenium nanoparticle conjugate and examination of its biological activity in vitro

**DOI:** 10.5599/admet.1023

**Published:** 2021-11-14

**Authors:** Sergey A. Staroverov, Sergey V. Kozlov, Alexander S. Fomin, Konstantin P. Gabalov, Vitaliy A. Khanadeev, Dmitry A. Soldatov, Ivan Yu. Domnitsky, Lev A. Dykman, Sergey V. Akchurin, Olga I. Guliy

**Affiliations:** 1 Saratov State Agrarian University, Saratov, Russian Federation; 2 Institute of Biochemistry and Physiology of Plants and Microorganisms, Russian Academy of Sciences, Saratov, Russian Federation; 3 Russian State Agrarian University - Moscow Timiryazev Agricultural Academy, Moscow, Russian Federation

**Keywords:** selenium nanoparticles, silymarin, bioavailability, cell lines

## Abstract

Silymarin (Sil) was conjugated to selenium nanoparticles (SeNPs) to increase Sil bioavailability. The conjugates were monodisperse; the average diameter of the native SeNPs was ~ 20-50 ± 1.5 nm, whereas that of the conjugates was 30-50 ± 0.5 nm. The use of SeNPs to increase the bioavailability of Sil was examined with the MH-22a, EPNT-5, HeLa, Hep-2, and SPEV-2 cell lines. The EPNT-5 (glioblastoma) cells were the most sensitive to the conjugates compared to the conjugate-free control. The conjugates increased the activity of cellular dehydrogenases and promoted the penetration of Sil into the intracellular space. Possibly, SeNPs play the main part in Sil penetration of cells and Sil penetration is not associated with phagocytosis. Thus, SeNPs are promising for use as a Sil carrier and as protective antigens.

## Introduction

Nanoparticles (NPs) are natural or chemically synthesized ultradispersed materials sized between 1 and 200 nm [1-2]. Various nanostructures, including polymers, dendrimers, liposomes, metal nanoparticles (Ag, Au, Ce, Cu, Eu, Fe, Se, Ti, Y, etc.), and silicon- and carbon-based nanomaterials have been successfully used as therapeutic agents and drug carriers [3–10]. The properties of NPs, such as their small size, large surface area, surface charge, chemical composition, and multifunctionality, make them unique drug carriers.

Much research attention has been paid to synthesized selenium nanoparticles (SeNPs). SeNPs have anticancer activity and are less toxic than Se salts [11-12]. SeNPs have been used as antimicrobial agents [13] and in the treatment of various diseases, including cancer, diabetes, inflammations, liver fibrosis, and drug-induced poisoning [14]. The main obstacle to the widespread use of Se is its low therapeutic index [15].

For example, Huang et al. [16] showed that small (5–15 nm) SeNPs are well able to scavenge free radicals. At < 0.5 mM, SeNPs had an excellent antioxidant effect. Compared to inorganic and organic Se compounds, SeNPs are more active biologically. However, their main disadvantage is their poor cellular penetration. Attempts have been made to solve this problem by conjugating the nanoparticles to various bioactive substances. This approach is a good foundation for cancer therapy. For example, SeNPs synthesized with quercetin and gallic acid have antioxidant, antimicrobial, and antitumor activities [17].

Surface ligands control the size and stability of SeNPs and improve their cancer selectivity, cellular uptake, bioavailability, and biological activity. The use of amphoteric ligands (polyethylene glycol, PEG) to make SeNPs has been described [18]. In another study, SeNPs were conjugated to a synthesized cyclic peptide that showed improved penetration into SK-OV-3 ovarian adenocarcinoma cells [19]. Consequently, SeNPs can be used as nanoscale delivery vehicles for differentially charged biomolecules and anticancer drugs. Specifically, SeNP coated with 5-fluorouracil (5-FU) exhibited increased anticancer activity in A375 cells [20].

SeNPs in combination with irinotecan increased the antitumor activity both *in vitro* and *in vivo* [11]. The combination of adriamycin and SeNPs proved a powerful approach to cancer chemotherapy. Low concentrations of this combination had a synergistic anticancer activity in Bel7402 liver cancer cells [21]. Antihepatocarcinoma effects were observed in HepG2 cells after the use of anisomycin-functionalized SeNPs, with NPs delaying the cell cycle in G0/G1 [22]. SeNPs (25 μg/ml) in combination with doxorubicin (2.5 μg/ml) showed a superior apoptotic effect in MCF-7 human breast cancer cells compared to the drug used alone. SeNPs also exhibit antitumor activity in in vivo-induced MCF-7 cells [23]. Biogenic SeNPs synthesized from an *L. plantarum* strain were immunostimulatory in BALB/c mice with breast cancer cells. Oral SeNP treatment significantly increased the production of proinflammatory cytokines such as IFN-γ, IL-2, IL-12, and TNF-α, and it also enhanced the delayed hypersensitivity response. SeNPs reduced tumor volume and increased survival in mice treated with SeNPs due to increased immune response [19].

Of particular interest is the use of SeNPs to increase the availability of drugs such as silymarin (Sil). Sil has a liver-protecting effect, reducing the concentration of free radicals and the degree of damage to the cell membranes, but its bioavailability is limited due to its poor water solubility [24-25].

The aim of this work was to conjugate SeNPs with Sil and analyze the biological activity of the resultant conjugate *in vitro*.

## Experimental

The conjugate was prepared by the following scheme: 0.39 g of Sil was dissolved in 30 ml of 0.1 M sodium hydroxide, and the solution was thoroughly mixed. Then, 2 ml of selenous acid from a 0.1 M solution and 1 ml of 0.03 M L-cysteine were added with vigorous stirring. The mixture was stirred for 1 h and lyophilized.

### Characterization of selenium nanoparticles

SeNPs size measurements were monitored by Dynamic Light Scattering (DLS) on the Malvern Zetasizer Nanoparticle Characterization System (Malvern Instruments, Great Britain) with the He-Ne laser (wavelength = 633 nm, power = 4 mW). The measurements were carried out at a fixed angle of 173° at 25 °C. The values of the hydrodynamic radius obtained by registering dynamic light scattering amounted to a peak of 25.2±1.5 nm for native gold particles.

SeNPs formations were monitored by spectrophotometry as described in [26]. Spectrophotometric evaluation of nanoparticles was carried out using a UV-Vis Specord BS 250 spectrophotometer (Analytik Jena, Germany) at a wavelength of 520 nm (A_520_). It is known that the maximum absorption of native nanoparticles was 526 nm, which is typical for particles with the property of surface plasmon resonance.

The average size, morphology and uniformity of the synthesized SeNPs also were studied by transmission electron microscopy (TEM) imaging as described in [26]. TEM images were recorded on a Libra 120 electron microscope (Carl Zeiss, Germany). Then these images were analyzed by ImageJ software to determine the morphology and size distribution of the SeNPs with a minimum of 100 nanoparticles measured.

The Sil concentration of the conjugates was measured by high-performance liquid chromatography at 288 nm, by using a Stayer chromatograph (Akvilon, Russia) fitted with a UV detector. Measurements were made with a 2.0 × 60-mm Luna C-18 column, with liquid-chromatography-grade acetonitrile: 1 % acetic acid solution (2:3, v/v) as the eluent.

### Biological activity of the conjugate

The biological activity of the conjugate was tested with the MH-22a, EPNT-5, HeLa, Hep-2, and SPEV-2 cell lines. The cells were cultured in Dulbecco’s modified Eagle’s medium (DMEM; BioloT) supplemented with 10 % fetal bovine serum (BioloT), penicillin (100 U/ml; Gibco), streptomycin (100 μg/ml; Gibco), and L-glutamine (292 μg/ml; Gibco).

Respiratory activity was measured conventionally [27] by the ability of cells to reduce nitrotetrazolium blue [3-(4,5-dimethylthiazol-2-yl)-2,5-diphenyl tetrazolium bromide, MTT (Sigma–Aldrich)] to formazan (MTT test). For each biological sample 10 replicates were analyzed.

### Affinity selection of miniantibodies from a phage library

Miniantibodies were selected from a phage library of sheep antibodies (Griffin.1), kindly provided by Professor W.J. Harris (Aberdeen University, UK) [28]. For the selection of phage carrying anti-Si l antibodies, a Western S membrane (size, 1 × 1 cm; Sigma Aldrich, USA) was used as a solid phase. The membrane was incubated overnight in a solution of the antigen (concentration, 1 mg/ml) at 4 °C. The antigen-coated membrane was then incubated in a 2 % solution of fat-free powdered milk for 30 min and placed in 1 ml of 10 mM TBS-T buffer (pH 7.2) containing the library phage at 10^12^ phage particles/ml. After the membrane was incubated overnight at 4 °C, it was washed five times with TBS-T buffer for 10 min. The bound phage was eluted with 7.18 M triethylamine (1 ml), and the pH was adjusted to 7.2 with 1 M TrisiHCl. The eluted phage particles were used to infect *E. coli* XL-1. The infected *E. coli* cells were grown overnight at 37 °C in 10 ml of 2YT liquid medium containing 100 μg/ml of ampicillin and 1 % glucose.

One liter of the 2YT medium contained 16 g of tryptone, 10 g of yeast extract, and 5 g of NaCl. A 1/100 portion (100 μl) of the resultant culture was inoculated into 10 ml of the 2YT medium, and the culture was grown in a thermostated shaker for 6 h until an absorbance (A600) of 0.3 (~10^12^ cells/ml) was achieved. This was followed by the addition of helper phage M13К07 and by incubation at 37 ℃ for 1 h. After incubation, the cells were sedimented by centrifugation at 2000 g for 10 min. The cell sediment was resuspended in 50 ml of the 2TY medium containing 100 μg/ml of ampicillin, 50 μg/ml of kanamycin, and 100 μg/ml of isopropyl-β-D-thiogalactoside and was grown overnight at 37 ℃ in a thermostated shaker. The overnight cell culture was again centrifuged at 3000 g for 40 min. To the supernatant liquid containing phage particles, a 1/5 volume of 20 % PEG 6000/2.5M NaCl was added, and the mixture was incubated on ice for 1.5 h. Phage particles were sedimented by centrifugation at 8000 g for 10 min, and the sediment was resuspended in 5 ml of TE buffer (1/10 the original culture volume; pH 7.5). The resultant preparation was clarified by centrifugation under the same conditions, and the phage particles were again precipitated by adding a 1/5 volume of PEG 6000/NaCl (1 ml) and were centrifuged. The sediment was suspended in 1 ml of TE buffer. The phage particle concentration was calculated spectrophotometrically using the ratio *A*_269_ = 30 ~ 2 × 10^14^ phage particles/ml.

The resulting phage particles were used for the next two rounds of selection. These were done under the same conditions but with a shorter incubation time and with fewer particles at the stage of their interaction with the immobilized antigen (1.5 h and 1 h of incubation at room temperature and 10^11^ and 10^10^ phage particles for the 2^nd^ and 3^rd^ rounds, respectively).

### Dot immunoassay

The specificity of the obtained phage antibodies was tested by dot immunoassay. A standard Sil solution was applied to the Western S membrane, and the membrane was blocked for 1 h with 2 % fat-free powdered milk in phosphate buffer. The membrane was dipped into a solution of specific phage, diluted to 10^13^ particles/ml of 10 mM phosphate buffer, and incubated at room temperature for 1 h. After that, the membrane was washed free from nonspecifically bound miniantibodies and dipped into a solution of colloid gold conjugated to rabbit antiphage antibodies (*A*_520_ = 0.5) [29].

### Phage labeling

The Sil-specific miniantibodies (1 × 10^12^ PFU) were resuspended in 100 ml of 0.3 M NaHCO_3_ solution (pH 8.6) containing 1 mg/ml of tetramethylrhodamine isothiocyanate. The reaction was carried out in the dark at room temperature for 1 h. After incubation, the volume of labeled phage was adjusted to 1 ml with PBS, and the phage was purified by dialysis. Finally, the fluorochrome-labeled phage was resuspended in 200 ml of phosphate buffered saline (PBS) and was frozen [30].

### Isolation of peritoneal macrophages

For peritoneal macrophages, the animals were killed and then fixed on their backs. An incision was made along the midline of the anterior abdominal wall, and the skin flap was carefully separated, with care taken to keep the peritoneum intact. After a puncture had been made with a needle connected to a syringe, 50 ml of PBS, pH 7.2, was injected into the peritoneal cavity. The anterior abdominal wall was then gently massaged, and after 5–7 min, peritoneal fluid was collected with a Pasteur pipet through a cut made in the peritoneum and filtered into a test tube through a nylon filter. The cells were washed three times by centrifugation in PBS at 750 g, after which they were redissolved in 1 ml of PBS and counted in a Goryaev chamber. Peritoneal macrophages were cultured by standard procedures [31], as described in Table 1.

For splenic lymphocytes, an incision was made along the white line of the peritoneum after peritoneal macrophages had been isolated, and the spleen was removed. The spleen was minced with scissors, and the tissue pieces were mashed through a fine sieve into a Petri plate containing sterile PBS. The resulting suspension was subjected to Ficoll–Urografin density-gradient centrifugation. The lymphocyte ring was collected into a new test tube. The lymphocytes were washed three times by centrifugation in PBS, pH 7.4, at 750 ×g for 10 min, and the cell pellet was redissolved in 1 ml of PBS. The lymphocytic cells were counted with a HaemaScreenvet hematology analyzer (Hospitex Diagnostics, Italy) [31], as presented in Table 1.

The cellTnanoparticle interaction was visualized on a DMLB fluorescence microscope (LEICA, Germany; excitation at 544 nm, emission at 570 nm).

The animals were cared for and handled in compliance with the requirements of the Ministry of Health of the Russian Federation (work of experimental biology clinics) and with the European Convention for the Protection of Vertebrate Animals Used for Experimental and Other Scientific Purposes.

### Statistics

Data were processed by the standard procedures integrated in Excel 2007 software (Microsoft Corp., USA).

## Results

The use of nano-Se enables researchers to make materials with improved physicochemical characteristics. In this context, many methods for preparing nanosized Se have been advanced [32,33]. These include green synthesis methods. Green synthesis is reliable, sustainable, and eco-friendly, and it avoids the production of unwanted or harmful byproducts [34]. Green synthesis of nanoparticles aims at minimizing waste and implementing sustainable processes. In recent years, green processes using mild reaction conditions and nontoxic precursors have been used in nanoresearch to promote environmental sustainability [34]. Contributions of researchers from different countries have led to remarkable progress in green synthesis. The versatility of green chemistry allows the preparation of a wide range of organic and inorganic nanomaterials with many promising applications [35]. The Green Analytical Procedure Index (GAPI) evaluates the green character of an entire analytical methodology, from sample collection to final determination. It was created with a tool such as the National Environmental Methods Index (NEMI) to provide general and qualitative information [36]. The existing methods are rated on the basis of four criteria that refer to the properties of reagents or wastes used in this method [37]. In this work, SeNPs were conjugated to Sil, and because the particles were reduced by using Sil (a milk thistle extract), we believe that according to the NEMI criteria, this method falls within green synthesis.

**Figure 1.**
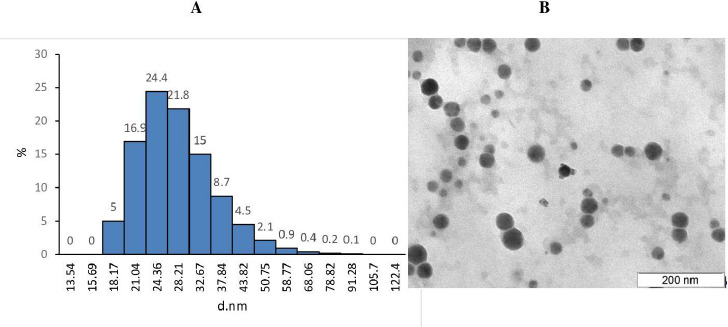
Size distribution of SeNPs, as found by DLS (**A**) and TEM (**B**).

After synthesis, it is very important to characterize the resulting nanomaterials carefully. The diameter of the synthesized SeNPs was measured by TEM and dynamic light scattering. Fig. 1 shows that the conjugates were monodisperse and that the average diameter of the native SeNPs was ~ 20-50 ± 1.5 nm with a peak ~ 25.2 ± 1.5 nm. The TEM results agree with the hydrodynamic diameter data. According to the literature, the most promising is the use of 20e70-nm SeNPs, the main advantage is their low toxicity, which permits them to be used in doses much greater than the daily requirement [33]. Therefore, the synthesized SeNPs were used for conjugation with Sil. The conjugate diameter was 30-50 ± 0.5 nm. High-performance liquid chromatography showed that the Sil concentration in the conjugate was 2 mg/ml. On this basis, the concentration of the conjugate was calculated for its further use in biological research.

**Figure 2.**
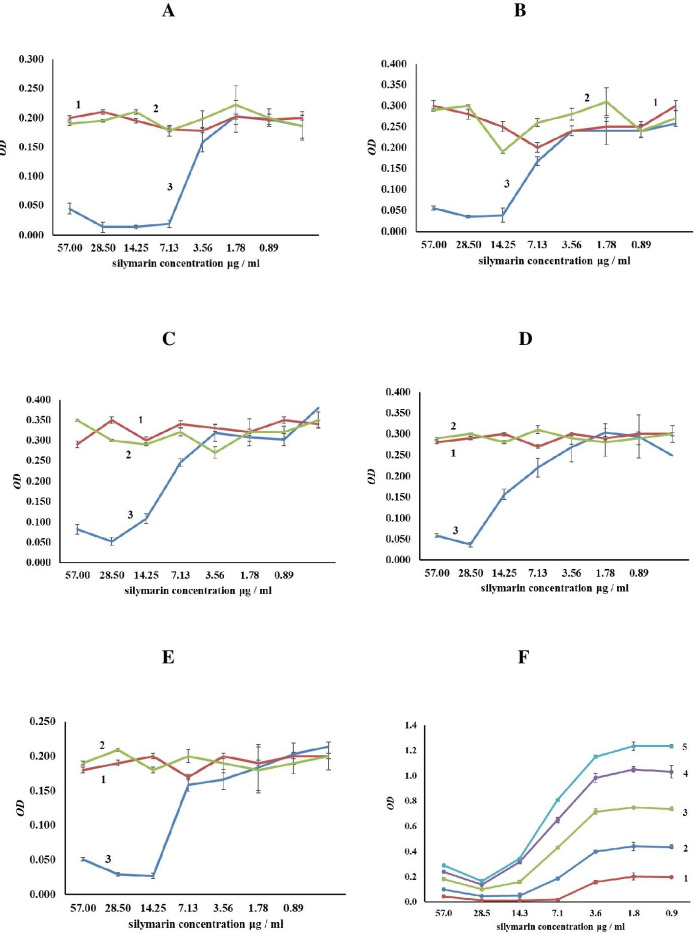
Changes in the cytotoxic effect of SeNPs/Sil on the cancer cell lines: EPNT-5 (**A**) HeLa (**B**); HEP-2 (**C**); MH22a (**D**); SPEV-2 (**E**): 1 – control (cancer cell lines were unexposed to SeNPs/Sil); 2 – cancer cell lines with Sil; 3 – cancer cell lines with SeNPs/Sil. (**F**) Total changes in the cytotoxic effect of the SeNPs + Sil preparation on various tumor cell lines without controls: 1 – EPNT-5; 2 – HeLa; 3 – HEP-2; 4 – MH22a; 5 – SPEV-2.

We next examined the cytotoxic effect of Se/Sil (Figure 2) on several cancer cell lines. For this purpose, the MH-22a, EPNT-5, HeLa, Hep-2, and SPEV-2 lines were treated with Se/Sil (Sil concentration, 0.89 57 μg/ml) for 24 h, and the change in absorbance was measured. There were two controls. In one of these, the cell lines were exposed to the above concentrations of Sil. In the other, the cell lines were unexposed to SeNPs or Sil. As presented in Fig. 2 (F), the most sensitive to the conjugate was the EPNT-5 glioblastoma line. Its activity decreased by 89 % compared to the control (cells grown without the conjugate). The Sil concentration, in this case, was 7.13 μg/ml. The other lines were sensitive to the higher 14.25 μg/ml Sil concentration. At 28.5 μg/ml of Sil, 85 % of the cells of the MH-22a line died.

**Figure 3.**
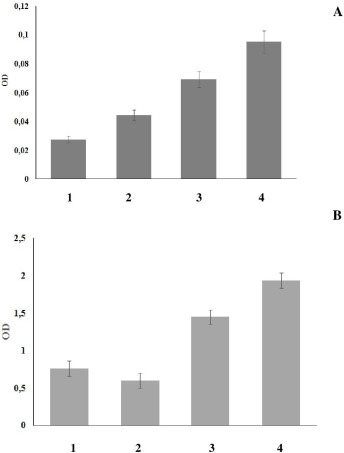
Changes in the respiratory activity of mouse peritoneal macrophages (**A**) and splenocytes (**B**) grown with Se/Sil: 1 – control (grown without SeNPs or Sil); 2 – grown with Sil; 3 – grown with Se; 4 – grown with Se/Sil.

We next investigated the effect of Se/Sil on the cells of the reticuloendothelial, or macrophage system because this cell system is responsible for the barrier, phagocytic, and metabolic functions. Fig. 3 (A) shows that the conjugate increased cell dehydrogenase activity by 3.4 times (*p* = 0.0003), as compared to Se alone, which increased cellular respiratory activity by 2.5 times (*p* = 0.0002). Fig. 3 (B) shows the effect of Se/Sil on mouse splenocytes. The conjugate increased cell dehydrogenase activity by 2.5 times (*p* = 0.0008) compared to Se alone, which increased cellular respiratory activity by 1.9 times (*p* = 0.0003).

**Figure 4.**
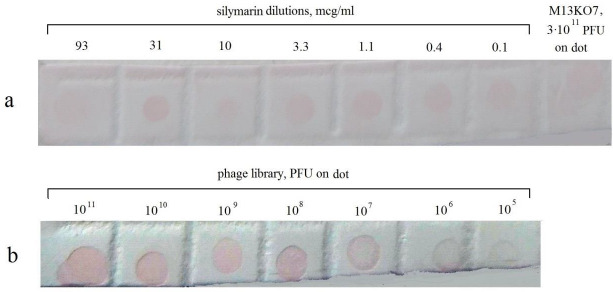
Dot immunoassay with selected SIL-specific antiphage antibodies (PVDF membrane, development with rabbit antibodies against the entire library, staining with protein A/colloidal gold). Anti-S mAb, titrated SIL (**a**); Library, application of phage library dilutions (**b**); Helper Phage, application of helper phage (10^12^).

Fluorescence microscopy was used to assess the interaction of Se/Sil with the cells of the reticuloendothelial system (macrophages and lymphocytes) of laboratory rats. The macrophages and lymphocytes were cultured as described in Materials and Methods. For visualization, we used antibodies prepared beforehand by phage display technology. The specificity of the phage antibodies was examined by dot immunoassay. Fig. 4 shows that the binding of the selected Sil-specific antiphage antibodies in the dot immunoassay was detected up to a Sil concentration of 0.1 μg/ml.

**Figure 5.**
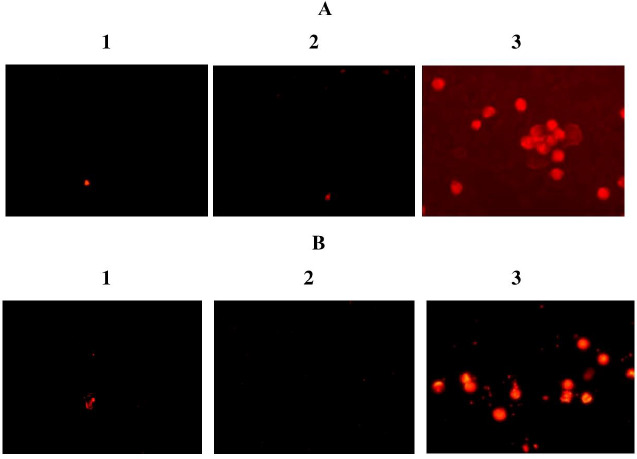
Visualization of the Se/Sil interaction with cells of the reticuloendothelial system [the macrophages **(A**) and lymphocytes (**B**) of laboratory rats]: 1 – control (cells cultured without Se/Sil); 2 – cells cultured with Sil; 3 – cells cultured with Se/Sil.

The fluorescence microscopy results for the interaction of Se/Sil with the cells of the reticuloendothelial system are shown in Fig. 5A for macrophages and in Fig. 5B for lymphocytes. The red fluorescence indicates the presence of Sil in the cells. Because there was no fluorescence when Sil acted alone, one can speculate that SeNPs are crucial for the Sil penetration of the cells. Probably, SeNPs facilitate the penetration of Sil into the intracellular space. Because fluorescence was observed when SeNPs were grown with lymphoid cells, the penetration of SeNPs is possibly unrelated to phagocytosis.

Thus, SeNPs/Sil is a promising anticancer conjugate that affects cellular immunity by causing the stimulation of both macrophages and splenocytes.

## Discussion

Nanomaterials, including SeNPs, can increase drug bioavailability. In this work, the bioavailability of Sil, used as an example, was increased by conjugation with SeNPs. Nanoparticles can penetrate cells by bypassing protective barriers (including the bloodrbrain and placental barriers) and can selectively accumulate in different parts of a living organism [2]. They can lead to inflammation, fibrosis, and cell and organ dysfunction, and they can also cause pathological disorders [38,39].

This situation imposes requirements for the development of adequate methodological approaches to the study of risks arising from the contact of biological systems with nanomaterials [40]. Therefore, any negative consequences should be analyzed before nanoparticle use, and risks should be weighed against any possible benefits. Although Se is a known antioxidant, it is a toxic element [41]. When choosing the chemical form of Se, one should pay attention to its effectiveness and safety. The most promising is the use of SeNPs, the size of which is 20m70 nm, because they are less toxic than other Se forms. Therefore, nanoparticles of this size can be used in doses that largely exceed the daily requirement [33].

In this work, 20I50-nm SeNPs were used to increase the bioavailability of Sil. The present study has shown promising prospects for the synthesis of SeNPs using silymarin. The EPNT-5 line was the most sensitive to the conjugate: the cell activity decreased by 89 % compared to the control. The conjugate increased cell dehydrogenase activity and promoted the penetration of Sil into the intracellular space. SeNPs are crucial to Sil penetration, and the process of penetration is unrelated to phagocytosis. In size range used, SeNPs are a promising platform for protective antigens and immunomodulators. The use of 20250-nm SeNPs avoids the toxicity of Se while increasing the bioavailability of Sil and possibly contributes to the anticancer treatment of the liver. The promise of using Se in cancer treatment has been repeatedly demonstrated [41]. The use of SeNPs in combination with Sil opens up the possibility of utilizing Se for liver cancer treatment. The obtained results are pioneering and will enable the range of anticancer drugs for the liver to be expanded.

## Figures and Tables

**Table 1.** Scheme for the growth of macrophages and lymphocytes with Se/Sil and the method of their staining with Sil-specific miniantibodies for subsequent microscopy

Group	Control	Sil solution	Sil/Se
Composition	10^6^ cells/ml in DMEM + HEPES with 10 % embryo serum	10^6^ cells/ml in DMEM + HEPES with 10 % embryo serum	10^6^ cells/ml in DMEM + HEPES with 10 % embryo serum
Step 1		Addition of a Sil solution (Sil concn, 1 μg/ml)	Addition of the Se/Sil conjugate (Sil concn, 1 μg/ml)
Step2	Incubate at 37 °C for 2 h, spin down the cells, resuspend the cells in a fresh medium		
Step 3	Fix aliquots of the cells on glass with acetone for 2 min		
Step 4	Apply phage (1012 per ml of PBS with 2 % BSA) for 1 h on both smears of each group; rinse twice with PBS		
Step 5	Rinse for 10 min with TrisRglycine buffer (pH 2.5); rinse twice with PBS; subject to microscopy (excitation at 544 nm, emission at 570 nm)		

## Abbreviations

DLS: dynamic light scattering
GAPI: green analytical procedure index
NPs: nanoparticles
NEMI: national environmental methods index
PBS: phosphate buffered saline
PEG: polyethylene glycol
PFU: plaque-forming units
SeNPs: selenium nanoparticles
Sil: silymarin
Se/Sil: Sil-linked SeNPs
TEM: transmission electron microscopy
UV: ultraviolet
